# Collectanea

**Published:** 1827-01

**Authors:** 


### COLLECTANEA.

Floriferis ut apes in saltibus omnia libant,
Omnia nos, itidem, depascimur aurea dicta.

### PHYSIOLOGY.

*Remarks on the Communication of the Lymphatic Vessels with the Veins.* By JOHN ROSSI, Doctor in Surgery, and Member of the Medico-Chirurgical Society of Bologna.—IN giving a short account of a series of researches on the lymphatic system, which I undertook this year in the great hospital of Parma, I am sensible that it is not in my power to enrich science with any important novelty, but merely to announce a few observations which may probably be useful in solving a physiological question.

It is known that many physiologists, having repeated those experiments which relate to the absorption of fluids, and having found many substances in the veins which they sought for in vain in the thoracic duct, have not hesitated to revive the ancient theory of venous absorption. It is, however, certain that various difficulties were successively pointed out against this doctrine; and that in two separate Numbers of the Anthology of Florence, in the course of the year 1824, it was announced that Dr. Regolo Lippi had discovered that many lymphatic vessels emptied themselves directly into the veins; and consequently, it being evident in what manner substances absorbed by the lymphatics were introduced speedily into the veins, that the experiments of Magendie, and others who maintained the doctrine of venous absorption, were overthrown, and became of no value. The importance of this question determined me to seek for these lymphatic trunks upon the dead body; and, although the work of Dr. Lippi has not yet been published, I do not think it necessary to retard the publication of my Remarks, since my object is not to refute any man's opinion, but merely to declare the kind of communication which I have been enabled to detect between the two systems.

Experiment 1st.—On the body of a young man who died of phthisis, in whom the abdominal viscera, as well as the mesenteric glands, were in a healthy state, I injected with mercury (according to the method of Walther) the lymphatic vessels passing out from the intestinal glands on the left side, after having tied the thoracic duct about four inches above the diaphragm. When a sufficient quantity of mercury had been thrown in, the thoracic duct below the ligature appearing to be full of the injected liquor, the operation was suspended. By the most accurate examination, I was not able to discover any lymphatic injected in the mesentery, and consequently I did not perceive any vessel emptying itself from the principal ramifications of the venæ portæ. I then removed the intestines, together with the internal lamina of the peritoneum which covers the lumbar portion of the vertebral column; thus exposing the aorta, the vena cava, and the lymphatic lumbar plexus, which was most beautifully injected. The vasa efferentia of the inguinal glands, in which the tube had been placed, after having passed the external and primitive iliac glands, went, as is usual, to the inferior lumbar glands, forming a plexus, and lastly to the superior lumbar glands: from these two the lymphatics went out, and, reuniting in a few trunks, gave origin to the receptacle of Pecquet. From the same glands I also saw three vessels take their origin, not very well filled with mercury, and not very small; which vessels, instead of going to the thoracic duct, emptied themselves evidently, one in the vena cava, just as it emerges from the posterior curvature of the liver, one at the beginning of the left emulgent vein, and the third again in the vena cava, near the termination of the right spermatic vein.

On the three points where these vessels united themselves with the above-mentioned veins, I passed a tight ligature; I then placed the injecting tube in the vasa inferentia of the lumbar glands, and soon after the three vessels became filled with the mercury. This mode of proceeding is always necessary whenever these trunks are required to be perfectly filled with the metal, in order to prevent its flowing into the veins. The mere appearance of these vessels induced me to suspect that they are lymphatics, although there is some little difference between them and the lymphatics which go to the thoracic duct, not only apparent to those accustomed to anatomical investigations, but also to the unpractised. The mere examination of their exterior does not appear to me sufficient to prove that they are lymphatics; and the more so, because they were never considered in that light by Mascagni, Rezia, Scarpa, Panizza, and many other celebrated anatomists; and I wished, therefore, to discover the truth by more certain methods,—that is, by examining their structure, and comparing them with those lymphatics concerning which there does not exist a doubt. I therefore removed from the body the three vessels just described, (which appear to be the same observed by Lippi, and recognised by him as lymphatics,) and, placing them upon a proper table, opened them longitudinally: I examined them with a good lens, and found that their internal surface was continuous, and that there was no indication of their possessing valves. I then took from the body some lymphatic vessels, and, opening them in the same way, I evidently saw valves throughout their whole length, which appeared to me to be double, and about two lines distant from each other.

We know that valves are constantly to be found in the lymphatic system of all animals. We also know that the venous ramifications of the three great cavities are wanting in valves, as well as all those whose diameters are less than one line; and therefore the valve is the great distinction between the lymphatic and the smallest blood-vessel. Besides this characteristic difference between the two vessels, it appears to me also that the thickness of the parietes, and the general aspect, as I have before said, was more like that of a vein than a lymphatic; since these latter vessels, when filled with mercury, present externally to the naked eye the knots produced by the valves, whilst those *supposed* lymphatics are merely cylindrical tubes, and, instead of knots, small interstices may be perceived between the globules of mercury, filled with a reddish liquor, which is in reality blood.

Finally, I separated from the body the lumbar and iliac glands, for other purposes foreign to these experiments, and found that, from the lateral and posterior surfaces of these glands, certain little vessels were given off, containing some globules of mercury; some of which went directly into the vena cava, others into the primitive iliac veins, and others into the last lumbar vein but one on the left side which traverses the lumbar column, passing under the aorta. These little vessels, which can only be seen by lifting up the glands, were a few lines in length, and there I could not examine them, but they had the aspect of veins.

Experiment 2d.—In the body of a youth, about eighteen years of age, which was emaciated to the greatest degree, I injected in the same manner the lymphatics of the mesentery. These vessels pursued their course to the mesenteric glands, and issuing from them again after visiting other glands, were reunited into a few trunks, which, passing near to the superior mesenteric artery, emptied themselves into the receptacle of Pecquet. In fact, the whole thoracic duct was full of mercury. From the above-mentioned glands, and particularly from the largest of them, vessels, containing but little mercury, having but a short course, proceeded, together with the lymphatics, and which terminated in the larger ramifications of the venæ portæ; many of them especially in the splenic vein. I was not able to find any of them terminating in the emulgent vein, as observed by Dr. Lippi. I made the same observations upon these vessels as in the preceding experiment, but I could not discover any indication of valves; whilst the lymphatics which emptied themselves into the thoracic duct were provided with them.

Experiment 3d.—The iliac lymphatics were injected in the body of a female, as in the first experiment, as well as those of the mesentery. These injections presented a greater number of branches, which emptied themselves into the vena cavæ, and into the ramifications of the venæ portæ. I obtained the same results on examining these branches, and comparing them with the true lymphatics, as in the former experiments.

Experiments 4th and 5th.—In two other extenuated bodies, I injected the iliac and mesenteric lymphatics. I obtained the same success, and demonstrated the same trunks as before. In one of the bodies, I began the injection above the right knee: the inguinal glands were injected, and I perceived that those minute venous ramifications which proceed from them, and go directly into the femoral vein prior to its passage through the crural arch, contained some globules of mercury, together with blood, and simulated the appearance of lymphatics. In order to be more certain that these little vessels possessed no valves, I put in practice the following plan:—I emptied the mercury from them by means of an incision made close to the glands from which they arose; then I placed the pipe in that point of the vessel in which they united with the veins, and scarcely had I opened the piston, when I saw the mercury run with velocity within these vessels, and make its exit at the aperture which I had made, which could not have happened had these been lymphatic vessels.

Experiments 6th, 7th, and 8th.—The iliac and mesenteric lymphatics of the bodies of three persons who had died of consumption were injected: the same branches going into the veins were perceived, and the most accurate examination afforded the same results as on the former occasions.

From these experiments it appears to me that the following deductions may be made:—

1st. That mercury injected into the lymphatic vessels, after having traversed the glands, passes into the veins by means of certain vascular branches, which afford a communication between the large veins and the glands themselves.

2d. That these branches, although having at first sight the appearance of lymphatics, ought to be considered as veins, whose principal office is to carry back the blood which is superfluous for the nutrition of those glands.

The mode in which I consider these vascular branches which pass from the glands to the veins, agrees perfectly with the ideas of Meckel and Fohman, who declare that the veins of the glands carry to the larger veins matters injected into the lymphatics. It also corresponds with the anatomical researches of Mascagni, who observes that, in many injections of the lymphatic system, the minute veins of the glands are filled, so as to appear like lymphatics; so that many authors before him were deceived by this appearance.

It being established, then, that the vessels seen in my experiments were veins rather than lymphatics, it is clear that the communication of vessels from one system to the other does not take place by branches; and it therefore only remains probable that it may be effected in the ultimate subdivisions of these vessels in the parenchyma of the glands. I say that this is probable, because the anastomoses in the interior of the glands have not been seen, and the argument is only upheld by the passage which the mercury makes into the venous ramifications; and, in truth, although injection evidently shows this passage, yet it may not exist in nature, because it may be the consequence of the impetus of the injection, concerning which it may not be useless to observe, that the lymphatic glands being provided with many blood-vessels, it may easily happen that the fluid injected into the lymphatics may open a road for itself into the veins: but this we may reasonably doubt, observing that the vessels passing out of the glands ought certainly to be destined to receive those substances which have been brought to them by the vasa inferentia.

On this point I think it may be proper to subjoin an observation, which may have some relation to this case. In a young female, who died of an affection of the chest, I found the lower extremities swollen and of considerable bulk. I would not use an injection, hoping to be able to see all the iliac and lumbar lymphatics; and I was not disappointed. I found them all full of lymph and well distended; the glands into which they went were loaded with lymph, from some of these, branches were seen which passed into the veins, and which had all the appearance of the vessels in question. These were all filled with a coloured matter, as were all the little veins. If a direct and natural communication had existed, it appears to me that the lymph would also have been found in these venous branches, although, after an accurate examination, I could not find any.

This discovery of the above-mentioned pretended lymphatics does not surprise me so much, (men of the greatest merit being sometimes deceived in making experiments,) as the following deduction which appears in the Anthology already quoted :—" This discovery, besides assuring to the lymphatics the exclusive faculty of absorption, substitutes truth and evidence to the hypotheses which have hitherto been formed for the purpose of explaining the rapid passage in the urine of certain matters taken with the food or as drink, and announced by the smell and colour."

Admitting for a moment the existence of true lymphatic trunks, which, arising from the intestines, empty themselves into the emulgent veins, ought we to believe that these veins deposit in the kidneys the substances absorbed from the internal surface of the intestinal tube? Are the emulgent veins canals that receive the blood from the cava, and carry it to the kidneys? Does the blood of the left spermatic vein, which discharges itself into the corresponding emulgent, go against its usual current to deposit itself in the kidney, rather than follow the course of the blood contained in the emulgent itself?

Such are the remarks which I proposed to make concerning the communication of these vessels. I do not pretend, however to have refuted by anticipation the work of Dr. Lippi, nor positively to conclude that no lymphatic trunk communicates directly between the two systems: I only wish to observe, that the branches seen by me, although at first sight they might seem to be lymphatics, must, after a rigid examination, be considered as veins; and it is a consolation to me to think that some consummate anatomists have expressed the same opinion. (*Annali Universali.*)

*Poisoned Wounds.*—At the Academie Royale de Medecine, M. Bouilland lately read a paper on some Experiments relative to the Effects of Compression in Poisoned Wounds. Nine experiments were made. In the first five, M. B. introduced two or three grains of strychnia into the cellular tissue of the thigh of a hare, so that he could at will use compression on the limb, either by a ligature above the wound, or with an unexhausted cupping-glass, or with the hand alone placed on the wound; and he found that he could, by applying the compression or not, either prevent or produce the effects of the poison. He found, by many trials, the good effects of compression in causing to cease or re-appear alternately the symptoms, according as he applied or removed the instruments.

In a sixth experiment, M. B. applied six leeches around the little wound, into which the strychnia was introduced; and he remarked that none of them would bite, yet they all died.

In the three last experiments, M. B. used half a tea-spoonful of hydrocyanic acid, instead of strychnia, and found the same results. He concludes—

1st. That these furnish new arguments in favour of the idea that many poisons are absorbed.

2d. That their deleterious effects would be prevented by hindering their absorption, or arresting the circulation in the part to which they are applied; and that it is in this way the cupping-glasses used by Dr. Barry act.

3d. That, in cases of poisoned wounds, a ligature above the wound would have the same effect as the cupping-glasses.

---

### PATHOLOGY.

*Variola.*—The following remarks contain nothing of novelty, but are of some interest, as showing the view taken by our continental neighbours.

From many cases of sporadic variola, M. Ribes, who makes the Reports of the Clinique of La Pitié, thinks that their severity depends entirely on the quantity of pustules. M. Bally divides cases of variola into three sorts—confluent, semi-confluent, and distinct; and his motives for so doing are these: The progress of semi-confluent is longer than that of distinct, and less than that of confluent; the pustules are more numerous than in the first, less so than in the second. We meet with agglomerated groups of pustules when all the rest are distinct; the pustules are less prominent than in the first form, but more so than in the second. The symptoms are more severe than in the distinct, less so than the confluent. In fine, the mortality differs essentially; for a much greater number of cases of semi-confluent are cured than of confluent, and almost all the cases of distinct are cured.

In those of the first class, which are very deadly, the most imminent danger is during the period of maturation; delirium, coma, enormous swelling of the face, extreme dyspnœa, great heat, frequent, full, and embarrassed pulse, &c. There is no morbid state, says M. Bally, where the symptoms appear more decidedly inflammatory, or where we are more imperatively called upon to use depletion, and yet where the bleedings, and other antiphlogistic means, are less efficacious. This remark is an additional motive for thinking that it is not the inflamed state of the skin, and the number of little phlegmons upon it, which constitute all the severity of the disease. In fatal cases, the inspection of the bodies has shown marks of death from true asphyxia, congestion of the brain and membranes, and considerable pulmonary engorgement. In some there were marks of pneumonitis; the larynx and trachea coloured of a reddish brown, and studded with variolous pustules, or rather traces of open pustules, the eruption scarcely ever passing the bifurcation: we have only once (says M. Ribes,) seen them about half an inch below it.

In the semi-confluent cases, which run through their course regularly, the period of desquamation is the most to be dreaded. Then the debility consequent to so severe an affection,—the irritation of the skin, and its denudation,—the greater "*impressionnabilité*" which remains, are often the sources of troublesome symptoms; such as very numerous subcutaneous abscesses, angina, irritations of the chest and intestinal canal, approaching to a state of phlogosis. (*Revue Medic.*)

---

### PRACTICAL MEDICINE.

*Peculiar Sore-throat affecting Children.*—The following observations are extracted from a paper in the Edinburgh Journal of Medical Science, by Dr. HAMILTON, jun. Professor of Midwifery.

In the last edition of the Hints for the Treatment of the principal Diseases of Infancy and Childhood, the following brief notice of an affection of the throat is inserted:—

"There is a very dangerous, but fortunately rare, modification of sore-throat, which begins in the form of a whitish spot, like that of thrush, (though more definite in its shape, being round or oval,) on one or both tonsils, unaccompanied at first by fever, and attended with only a trifling degree of uneasiness in swallowing. By and by this spot enlarges, its edges become of a florid colour; fever steals on; and the act of swallowing grows painful. A slough gradually forms, with evident ulceration at its edges; the fever increases; and headache and restlessness supervene.

"The partial separation of the slough, together with the rosy colour of the edges of the ulcer, with the moderate degree of fever for some days, promise a favourable issue. But very unexpectedly slowness of breathing, without either difficulty or wheezing, takes place, with excessive and sudden sinking of the living powers; and it generally happens that, within a day from this change, the fatal event takes place. The breathing at first falls to eighteen respirations in the minute,—then to sixteen,—to twelve,—and finally to ten or eight. Sometimes, with the sloughing, the tonsil swells; and in some cases both tonsils are affected.

"Hitherto, with one exception, this disease has proved mortal in every case in which the author has been consulted; and he considers the slow breathing to be a sure symptom of the fatal termination.

"With respect to the nature of the disease, his experience is too limited to enable him to give a decided opinion. He has repeatedly known two individuals of the same family attacked in succession with the disease, but he does not feel warranted in pronouncing it to be infectious.

"It is with feelings of sincere regret that he has to state that no mode of treatment, yet discovered, seems to have any influence in checking the progress of this disease. The most powerful local applications, such as stimulating gargles, the use of caustic, &c , and all the ordinary means of supporting the strength, have, in the cases to which the author was called, been pursued with much anxiety and activity, without any benefit. The operation of opening the windpipe was, in one interesting case, (where, from the swelling of both tonsils, there was apparently a mechanical obstruction to the breathing,) had recourse to without any avail."

Further experience has convinced the author of these remarks that two other symptoms occasionally attend the disease. The one is a most offensive fetor of the breath, and the other is the sudden occurrence of cynanche trachealis. The former of those symptoms has not been perceptible in the cases he has attended, or on which he has been consulted, sooner than from the sixth to the ninth day after the disease had been distinctly marked; and the latter has hitherto occurred before the sixth day.

Thus this very curious affection proves fatal in two different ways,—viz. by exciting cynanche trachealis, or by inducing slow breathing and progressive sinking of the living powers; and, in repeated instances, these two different terminations have occurred in the same family.

There can be no difficulty in understanding the former of these symptoms; for it is evident that the local inflammation of the tonsils and uvula is, in such cases, extended to the larynx, after having affected the pharynx.

But the other, and (as far as the author's personal observation warrants him to believe) the more common termination, does not admit of such a ready explanation. The partial separation of the slough, the rosy colour of the edges of the ulcer, and the moderate degree of fever, make the slow breathing, which suddenly supervenes, very unexpected to the attendants; and the rapidity with which death follows the slowness of breathing has appeared quite wonderful. The explanation which occurs to the author is, that the matter secreted by the ulcer, being evidently of the nature of a morbid poison, may act by paralysing, or otherwise influencing the par vagum, or the branches of the eighth pair of nerves of the medulla oblongata, on which Le Gallois has proved, by direct experiment, that breathing depends.

(Here follows some reasoning upon the probability of absorption.)

If the reasoning thus offered be correct, it should follow that, in all cases of ulceration on surfaces communicating with the respiratory nerves, the physician, in the treatment of the disease, should, by the most vigorous efforts, endeavour to accomplish two objects,—viz. to prevent the extension of the local inflammation, and to lessen the diseased secretion.

The former indication seems, at first sight, the more important one in the particular and curious disease now under consideration; for it appears, for some time after the attack, to be merely a local affection. Accordingly, means calculated to fulfil this intention were suggested, and pursued with great energy, in all the cases till lately to which the author was called. He was, however, mortified to find that neither the application of leeches, followed up by purgatives and blisters, nor the use of topical stimulants,—such as the various irritating gargles, the application of the nitrate of silver, and even scarifications, proved in the slightest degree beneficial. He was resolved, therefore, to take the first opportunity of ascertaining the efficacy of attending principally to the second indication: that opportunity has lately occurred, and he now communicates the result of the experiment.

On Tuesday, June 27, 1826, he was requested by Dr. Torrance (an old pupil) to visit a little boy, aged six years, labouring under a modification of sore-throat, which appeared to Dr. Torrance to be of a singular character. The Doctor stated that this was the third child in the family who had become affected with the disease. The first of these was a girl, nine years old, still alive, but apparently sinking. She had been ill since the 12th day of June; and, ten days after her attack, her brother, aged four years, was seized with the same symptoms, and, after a certain progress, cynanche trachealis supervened, and proved fatal on the 26th of June; so that this little boy was only four days ill.

Before visiting the patient, the description contained in the last edition of the Hints for the Management of Infants and Children was put into Dr. Torrance's hands, and he immediately declared that it applied most accurately and minutely to the cases he had been attending, with a single exception,—viz. that the excessive fetor from the ulceration of the throat was not specified in the printed description.

When the state of the little boy was examined, the slough upon each tonsil, larger upon the right than on the left, with a slight swelling of the velum, was strongly marked, but there was no fever. It was ascertained that the first symptom of indisposition had been discovered on the preceding morning, and that he had been treated with great activity,—having had a dose of calomel and jalap, which had operated both upwards and downwards, and having had leeches applied externally to the throat. Besides the attendance of Dr. Torrance, the little patient had the benefit of the assistance of two intelligent young gentlemen studying medicine, who lodged in the house; and they, by the by, cordially concurred with Dr. Torrance in bearing testimony to the accuracy of the coincidence between the printed description of the disease, and the progress of the symptoms in the children they had attended. The state of the little girl was desperate, and she sunk on the 30th of June.

Agreeably to the views already explained, it was suggested that the great object to be aimed at should be to lessen the diseased secretion from the throat, and with this intention the sulphate of quinine, in as large doses as could be administered, was prescribed. At the end of twenty-four hours it was found, however, that this medicine could not be retained, or rather, perhaps, could not be received in sufficient doses; and, as the ulceration was increasing and fever was stealing on, it became necessary to change the medicine without altering the indication.

Adverting to the extraordinary efficacy of the sugar of lead in restraining passive hemorrhagy, and in lessening secretions depending upon laxity of fibre, it was judged that this medicine might answer the purpose in view, and at any rate that it merited a fair trial. Eight grains dissolved in eight ounces of rose-water, to which forty drops of tinctura opii were added, were therefore prescribed; and of this half an ounce were directed to be given every three hours while awake. Instead of the stimulating gargle hitherto employed, consisting of capsicum, &c. a solution of the sugar of lead, of the strength of a scruple to eight ounces of distilled water, was substituted. A dose of castor-oil was directed to be given every second morning.

Under this treatment, from which no inconvenience whatever was experienced, the sloughs gradually contracted, and the swelling of the velum pendulum palati progressively decreased, in the course of which it was discovered that the ulceration had extended over the pharynx behind the velum, and the cure was completed on the 16th of July. The medicine was begun on the 28th of June, and was continued till the 14th of July, occasionally diminishing and increasing the intervals between the doses. The whole quantity of the acetate taken during that time was twenty-four grains.

From the cases of this disease which have fallen under the author's notice, he is now inclined to believe that it is infectious. While he has repeatedly seen two children in one family attacked in succession with the disease, he has only known one instance where, in a family of several children, it was confined to a single individual. He may add that, in so alarming and fatal a malady, he considers it the duty of a physician to hold out that it is infectious, and to enforce all the ordinary precautionary measures to prevent its spreading, even although certain doubts of its infectious nature might impress his own mind.

The remarks in the preceding pages are offered in illustration of a general principle applicable to cases of morbid ulceration, which an intelligent practitioner can modify according to the individual instances falling under his charge.

That remedies must be adapted not only to the particular constitutions of the patients, but also to the complications and the degrees of severity of the symptoms, is a proposition generally assented to, but not always acted upon; and yet it is in this art that the talents of an active and intelligent practitioner are chiefly conspicuous. This consideration should lead every physician to avoid forming a prejudice in favour of one or two drugs for the cure of particular diseases. Thus, in some cases of the disease now under notice, the sulphate of quinine or of zinc, or the ammoniarate of copper, or some of the oxides of iron, or the mineral or vegetable acids with wine, or even some of the preparations of mercury,* may be preferable to the sugar of lead; but the facility and safety with which that latter medicine can be given, makes it a most convenient prescription for children. In cases of cynanche maligna, a gargle composed of a solution of this medicine might probably be substituted, with much advantage, for the stimulants hitherto employed.

*Tincture of Cantharides for the Bites of Venemous Reptiles.*— M. le Docteur Ricord, Sen. during a long residence in America discovered what he considers a sure mode of preventing mischief from such bites. "It is sufficient (he says) to pour a few drops of

* In the only case of cynanche trachealis supervening to the cynanche maligna which the author has seen terminate favourably, the boy (four years old) had a dose of calomel every hour while the symptoms continued violent, and at the same time was allowed wine in large quantities.

tincture of cantharides on the wound to cause a redness and vesication; not only is the poison rendered harmless, but the stings of the reptiles are removed with the epidermis that the blister raises." —*Revue Medicale.*

*Laurel Water in Epilepsy, by* Dr. MULLER.—A young girl, twenty-two years of age, had been epileptic for six years. The attacks frequently returned twice in one day; they were of short duration, and in the intervals the patient had spasms in the arms, and moved her fingers in a convulsive manner. She had been for eighteen months bedridden, unconscious of her state and actions, eating and drinking any thing offered, but asking for nothing, and passing her stools involuntarily. A variety of means were tried without effect, and all of them, but particularly large bleedings, appeared to be hurtful. M. Muller was called, and found the patient in this state. She had always been regular in menstruating, and had never had any but the ordinary diseases of infancy, and never any chronic eruptions of the skin, nor worms. The tongue was soft and moist, belly pliant, respiration natural. Not being able to find any cause for this disease, M. M. thought of employing laurel water, from which he had often derived great advantages in nervous affections similar to this. He prescribed it in the dose of twenty drops daily. After the consumption of an ounce, the convulsive movements of the limbs had completely ceased; and after the administration of three ounces more, (augmenting each dose two drops, till the dose was eighty drops,) the attacks of epilepsy never returned. The patient having recovered her sensibility, left her bed, and executed spontaneously every function. The treatment was concluded by an infusion of Valerian, with addition of Træ. Canellæ and Liquor Ammoniæ; and, after using for some time some preparations of iron, she quite recovered her health. (*Ibid.*)

*Cold Water in Tic Douloureux.*—Dr. BIRD, in one of the German Journals, relates a case where a lady, whose sister had fallen a sacrifice to the agonies of tic douloureux, was herself, six weeks after her first lying in, seized most severely with this disease. Many physicians, and of course many remedies, were tried, but in vain, till Dr. Bird suggested the application of compresses dipped in cold water, and applied to the pained part. This immediately had the desired effect, and always afterwards relieved her in future attacks!

*Intermittents.*—In these fevers M. BALLY freely uses the sulphate of quina, in doses carried to the extent of twelve to sixteen grains, and then gradually decreasing them when the paroxysms have ceased to appear. M. B. thinks that it is both useless and dangerous to wait to prepare the patient for the administration of the remedy; he recommends immediately attacking it by the doses of quina above mentioned. Severe and obstinate headache ordinarily follows the sudden stopping of an intermittent of some duration; it appears to be occasioned by the nervous derangement which has so great a share in these affections: the subcarbonate of iron, gradually increased, appears to relieve it. M. Bally (it is said) has at last proved the important fact, that sulphate of quina even in large doses always retards and softens the pulse. (*Ibid.*)

*Exhibition of Sulphate of Quina by Friction.*—M. BALLY, having observed that the sulphate of quina very frequently irritates the stomach when swallowed, determined to try its effects by rubbing it on the gums and the mucous surface of the lips. He cured nine cases of intermittent and remittent fevers by this method: the dose was usually from four to eight grains morning and evening. The only inconvenience was from the extreme bitterness of the medicine, which he suggests might be corrected by mixing some other substance with it.

---

### SURGERY.

*Injury of the Rectum.*—A nurse, in applying a glyster, introduced the point of the syringe so roughly and unskilfully as to push it through the back part of the rectum. By the exertion of considerable force, she emptied the contents of the syringe into the pelvis. The patient, a young lady, suffered considerable pain afterwards. On the sixth day a membranous mass passed away with the fæces, which was ascertained to be a portion of the rectum. Upon examination, an opening was found in the back part of the gut, about the size of a dollar, and about two inches from the anus. If a sufficient quantity of water was injected, the rectum was distended, and also the interval between the posterior surface of the gut and the sacrum, in consequence of the fluid escaping through the aperture, the edge of which could be felt loosely floating. The greatest distress and danger arose from the escape of the fæces into the pelvis. Injections were used for the purpose of washing out any portion which passed through the opening: a part of the fæces still passed in the natural manner. Mild injections were frequently used, in a small quantity, that the rectum might not be kept separated from the sacrum. Light broths, yolk of eggs, &c. were allowed as diet. Professor GRAFE was consulted under these circumstances, and he adopted the following plan. He introduced a portion of the intestine of an animal into the rectum, and, having filled it with water, he tied the end projecting from the anus: by this means the rectum was kept in contact with the sacrum. Air was afterwards substituted for water, as the weight of the latter was found inconvenient. This plug was removed every twenty-four hours, and the fæces, which its presence prevented from descending, evacuated; and the intestine well oiled, again introduced, and filled with air. A gradual improvement followed, and in a few weeks the wound was entirely closed, without any contraction of the rectum, and the fæces were passed without difficulty.—*Journal fur Chirurgie von Gräfe und Walther, band* ix.

*Acupuncturation in Tic Douloureux.*—Two cases are given by Dr. Bergamaschi, illustrating the good effects of acupuncturation in this most painful disease, when most other known remedies had been tried, and failed. J. Crespi, æt. 38, born of healthy parents, himself of good constitution, and always healthy, had occasion to work for some time in a damp place, and soon afterwards a severe and almost insupportable pain came on in the occiput, behind the ear, along the zygomatic apophyses, in the upper jaw, in the forehead, and particularly over the eyebrow. During five months every means were tried; as the use of camphor, musk, belladonna, hyoscyamus, stramonium, opium, leeches, blisters, mercury, electricity, but all without success. A convulsive twitching agitated the right eyelid and upper lip. M. B. ordered leeches, and, after their application, neutral salts, with tartarised antimony. This calmed the patient, but next day violent paroxyms came on, and two days after the patient was so ill as to threaten to destroy himself if not relieved. A steel needle was plunged during a slight remission into the superior and anterior part of the temple, and left for a few minutes; another, introduced near the angle of the inferior jaw, penetrated to the bone; a third pierced the massetic muscle; and the last was placed running from before backwards in the mastoid apophysis towards the angle of the jaw. They were left in only during ten minutes, before the expiration of which the pain had ceased. Some pain having returned on the following days, it was necessary to have recourse from time to time to the acupuncturation, which always removed it as if by enchantment. Soon afterwards the patient resumed his occupation, and has had no return.

Martin Pasca, æt. 43, very robust, after working with his arms exposed on the morning of January 8, 1825, was suddenly seized with sharp pain in the vault of the palate, extending to the tongue, and externally to the right cheek, to the masseter, and towards the angle of the inferior jaw, occupying also the ear, the muscles of the neck, and the integuments. For four months the pains were so dreadful that, from fear of increasing them, the patient generally refused to eat or drink ; he became emaciated in consequence. When called to this case, M. B. first ascertained whether any carious teeth might have caused the disease; which not being the case, he plunged transversely into the buccinator muscle a long steel needle, a second behind the ear, and a third at the angle of the inferior jaw ; they were allowed to remain for eight minutes, during which time the convulsive contractions of the lower lip, as well as a great part of the pain, ceased, and the patient afterwards had some sleep. This relief did not last long: a meal taken in the evening brought back all his sufferings, which were next morning removed by the introduction of five needles, left in for twelve minutes; before using these last, M. B. had given an emulsion, containing Ext. Hyoscyami: the pain ceased suddenly, and was succeeded by a sense of itching. From this time the exhibition of Ext. Hyoscyami internally, and frictions with oil mixed with this substance, were the means used, and during eight months scarcely the least return of pain was experienced.

